# Supplementary material for: In vitro fish mucosal surfaces producing mucin as a model for studying host-pathogen interactions
Source: PLoS One. 2024 Aug 9;19(8):e0308609. doi: 10.1371/journal.pone.0308609 (PMC11315345; doi:10.1371/journal.pone.0308609)
Supplement: S1 Raw image — (PDF) [file pone.0308609.s001.pdf]

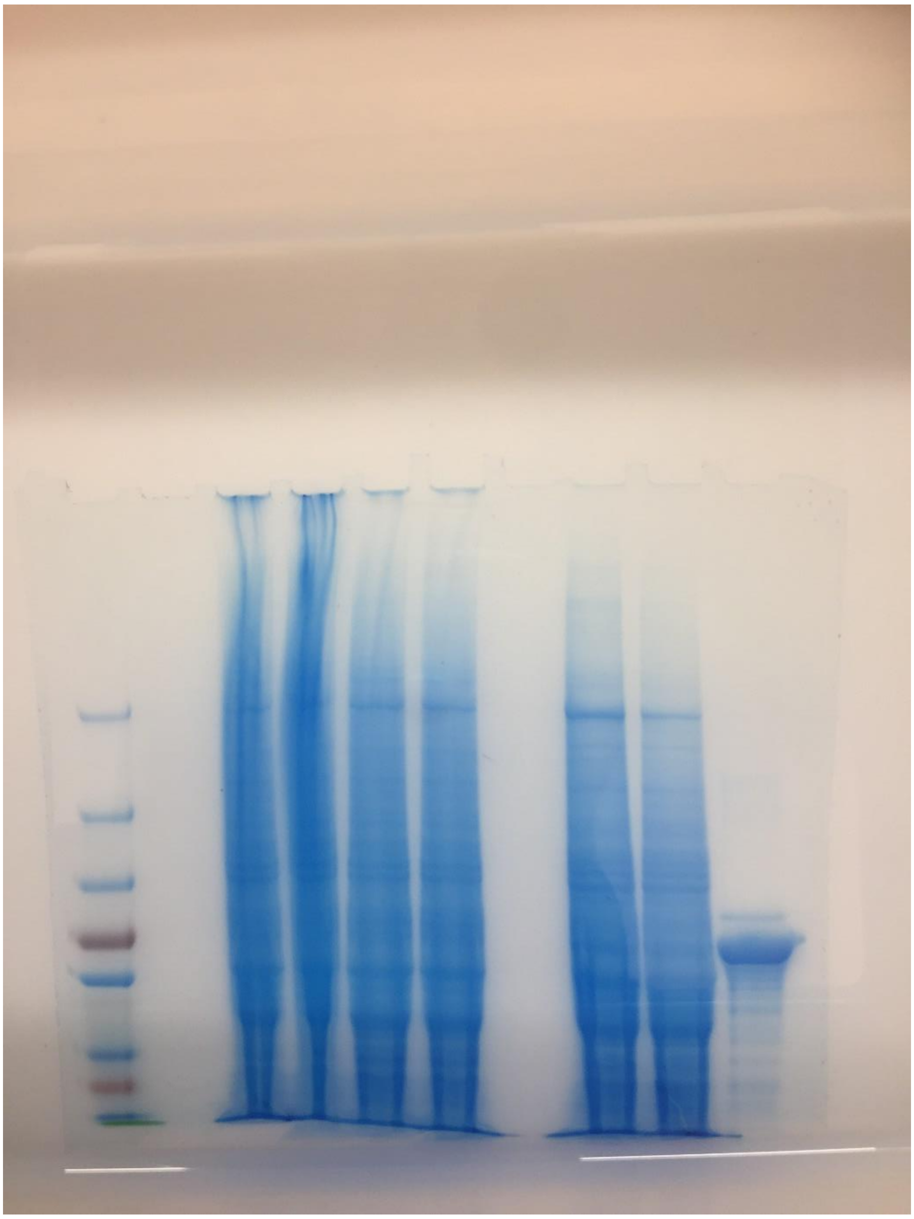

S1 Fig. The uncropped image of the gel from Figure 3. *O*-glycans on high molecular weight glycoproteins from cell lysates of cell lines CHSE214 and RTGill-W1. Cell lysates were analysed on 3-8% tris acetate gels, and developed with Alcian Blue, which stains acidic glycoproteins. Lane 1 (to the left) contains the molecular weight marker: the top bans is 250, the second 130, the third 100 and the fourth 70 kDa. The Lanes 3-6 (from the left) contain CHSE-214, lanes 7 and 8 RTGillW1 and lane 9 cell culture media. Lane 2 and 7 are empty.
