# Supplementary material for: In vitro fish mucosal surfaces producing mucin as a model for studying host-pathogen interactions
Source: PLoS One. 2024 Aug 9;19(8):e0308609. doi: 10.1371/journal.pone.0308609 (PMC11315345; doi:10.1371/journal.pone.0308609)
Supplement: S1 Table — (DOCX) [file pone.0308609.s002.docx]

**S1 Table 1.** **Fluorescence intensity dataset for Figure 2.**

| RTgill-W1 | CHSE-214 |
| --- | --- |
| \| 17.3 \| \| --- \| | 68.6 |
| \| 36.1 \| \| --- \| | 39.7 |
| 30.1 | 43.9 |
| 36.3 | 18.4 |
| 47.5 | 53.7 |
| 35.2 | 76.4 |
