# Supplementary material for: In vitro fish mucosal surfaces producing mucin as a model for studying host-pathogen interactions
Source: PLoS One. 2024 Aug 9;19(8):e0308609. doi: 10.1371/journal.pone.0308609 (PMC11315345; doi:10.1371/journal.pone.0308609)
Supplement: Attachment — Submitted filename: Reviewers comment_PlosOne.docx [file pone.0308609.s003.docx]

**Manuscript Number:** PONE-D-23-19772

**Full Title:** Mucin Producing In vitro Fish Mucosal Surfaces as a Model for Studying Host Pathogen Interactions

**Reviewer’s comments**

1. Use full form of RT and mention room temperature for country where the experiment was performed. (Line no – 194)
2. Typographical error in writing 14000 kD. (Line no – 224)
3. Typographical error in writing 4 °C. (Line no – 228)
4. Ultimate3000, is this correct way to write or typographical error? (Line no – 236)
5. autosampler, is this correct way to write or typographical error? (Line no – 236)
6. Typographical error in writing 35 %. (Line no – 244)
7. Typographical error in writing 20 °C. (Line no – 257)
8. OD600nm, is this correct way to write or typographical error? (Line no – 268)
9. Use standard format while writing Alcian blue in the entire manuscript.
10. interindividual, is this correct way to write or typographical error? (Line no – 337)
11. nonderivatized, is this correct way to write or typographical error? (Line no – 349)
