# Supplementary material for: In vitro fish mucosal surfaces producing mucin as a model for studying host-pathogen interactions
Source: PLoS One. 2024 Aug 9;19(8):e0308609. doi: 10.1371/journal.pone.0308609 (PMC11315345; doi:10.1371/journal.pone.0308609)
Supplement: Attachment — Submitted filename: Response to reviewers cell line.docx [file pone.0308609.s004.docx]

**RESPONSE to REVIEWERS and EDITOR**

We thank the editor and reviewers for providing many useful suggestions on how to improve our manuscript (new title: *In vitro* fish mucosal surfaces producing mucin as a model for studying host-pathogen interactions, PLOS ONE, PONE-D-23-19772). We have addressed all of the issues raised by the reviewers in our revised manuscript with a detailed description of each. We trust that the manuscript is now suitable for publication in PLoS ONE. Thank you for your continued consideration of this manuscript.

Sincerely,

Sara Lindén

Response to the EDITOR

1. Please ensure that your manuscript meets PLOS ONE's style requirements, including those for file naming. The PLOS ONE style templates can be found at 
<https://journals.plos.org/plosone/s/file?id=wjVg/PLOSOne_formatting_sample_main_body.pdf> and 
<https://journals.plos.org/plosone/s/file?id=ba62/PLOSOne_formatting_sample_title_authors_affiliations.pdf>

Response: The manuscript has been formatted according to the guidelines, including title, author and affiliations, references, and manuscript body. The formatting changes are highlighted in yellow in the revised manuscript.

2. Please note that funding information should not appear in any section or other areas of your manuscript. We will only publish funding information present in the Funding Statement section of the online submission form. Please remove any funding-related text from the manuscript.

Response: We have removed the funding information from the manuscript.

3. We note that your Data Availability Statement is currently as follows: "All relevant data are within the manuscript and its Supporting Information files. Raw MS data on O-glycan analyses are available on glycopost ( <https://glycopost.glycosmos.org/>) using the project ID: GPST000334 (CHSE-214) and GPST000335 (RTgillW1). MSMS data with tentative structures are available <https://unicarb-dr.glycosmos.org/references/523>."

Please confirm at this time whether or not your submission contains all raw data required to replicate the results of your study. Authors must share the “minimal data set” for their submission. PLOS defines the minimal data set to consist of the data required to replicate all study findings reported in the article, as well as related metadata and methods (<https://journals.plos.org/plosone/s/data-availability#loc-minimal-data-set-definition>).

For example, authors should submit the following data:

- The values behind the means, standard deviations and other measures reported;
- The values used to build graphs;
- The points extracted from images for analysis.

Authors do not need to submit their entire data set if only a portion of the data was used in the reported study.

If your submission does not contain these data, please either upload them as Supporting Information files or deposit them to a stable, public repository and provide us with the relevant URLs, DOIs, or accession numbers. For a list of recommended repositories, please see <https://journals.plos.org/plosone/s/recommended-repositories>.

If there are ethical or legal restrictions on sharing a de-identified data set, please explain them in detail (e.g., data contain potentially sensitive information, data are owned by a third-party organization, etc.) and who has imposed them (e.g., an ethics committee). Please also provide contact information for a data access committee, ethics committee, or other institutional body to which data requests may be sent. If data are owned by a third party, please indicate how others may request data access.

Response: All data are available. We have made a supplementary file to which we added the original photo of the gel from Figure 3 and the datapoints forming the basis for the graph in Figure 2. In addition, the MS data which can be accessed on <https://glycopost.glycosmos.org/> using the project ID: GPST000334 (CHSE-214) and GPST000335 (RTgillW1) and MSMS data with tentative structures on available <https://unicarb-dr.glycosmos.org/references/523>."

4. When completing the data availability statement of the submission form, you indicated that you will make your data available on acceptance. We strongly recommend all authors decide on a data sharing plan before acceptance, as the process can be lengthy and hold up publication timelines. Please note that, though access restrictions are acceptable now, your entire data will need to be made freely accessible if your manuscript is accepted for publication. This policy applies to all data except where public deposition would breach compliance with the protocol approved by your research ethics board. If you are unable to adhere to our open data policy, please kindly revise your statement to explain your reasoning and we will seek the editor's input on an exemption. Please be assured that, once you have provided your new statement, the assessment of your exemption will not hold up the peer review process.

Response: The data is already uploaded on <https://glycopost.glycosmos.org/> using the project ID: GPST000334 (CHSE-214) and GPST000335 (RTgillW1) and <https://unicarb-dr.glycosmos.org/references/523>. Once the manuscript is accepted it is a matter of minutes to make the data publicly accessible.

5. We note that you have included the phrase “data not shown” in your manuscript. Unfortunately, this does not meet our data sharing requirements. PLOS does not permit references to inaccessible data. We require that authors provide all relevant data within the paper, Supporting Information files, or in an acceptable, public repository. Please add a citation to support this phrase or upload the data that corresponds with these findings to a stable repository (such as Figshare or Dryad) and provide and URLs, DOIs, or accession numbers that may be used to access these data. Or, if the data are not a core part of the research being presented in your study, we ask that you remove the phrase that refers to these data.

Response: we have removed the comments pertaining to the cell line that did not adhere as we did not take any photos of the empty plastic surface.

We also removed the sentence “We tested labelling of mucins at 4 h post addition of GalNAz, however the label was not yet incorporated into the cells in sufficient amounts to be easily detected (data not shown).”

6. PLOS ONE now requires that authors provide the original uncropped and unadjusted images underlying all blot or gel results reported in a submission’s figures or Supporting Information files. This policy and the journal’s other requirements for blot/gel reporting and figure preparation are described in detail at <https://journals.plos.org/plosone/s/figures#loc-blot-and-gel-reporting-requirements> and <https://journals.plos.org/plosone/s/figures#loc-preparing-figures-from-image-files>. When you submit your revised manuscript, please ensure that your figures adhere fully to these guidelines and provide the original underlying images for all blot or gel data reported in your submission. See the following link for instructions on providing the original image data: <https://journals.plos.org/plosone/s/figures#loc-original-images-for-blots-and-gels>. 
  
In your cover letter, please note whether your blot/gel image data are in Supporting Information or posted at a public data repository, provide the repository URL if relevant, and provide specific details as to which raw blot/gel images, if any, are not available. Email us at [plosone@plos.org](mailto:plosone@plos.org) if you have any questions.

Response: We have included the original uncropped photo of the gel shown in Figure 3 as supplementary Figure 1 and added (The uncropped image is available as Supplementary Figure 1) to the figure legend of Figure 3.

Review Comments to the Author

Reviewer #1: This manuscript describes an interesting novel in vitro model based on modified fish cell lines for the study of mucin responses in mucosal epithelia. Its reading was inspiring and it will help to develop alternative methods to the in vivo ones.
There are some suggestions authors could follow in order to detail results and deepen discussion, which in my opinion could increase the study’s soundness.
-I wonder what the cell morphology in the in vitro membranes was, and if it changed after DAPT treatment. Could authors observe goblet cell morphology or cell polarization? Including a description on cell morphology, differentiation and polarization would be interesting. In fact, authors have included in the discussion section the issue of non-differentiation and non-polarization in unstimulated cultures on plastic surfaces, leaving this issue unanswered in the current model.

Response: We do not have data pertaining to this topic with the exception of the images provided. Therefore we removed the sentence mentioning polarization and apical from the discussion, although we did not claim that the current model had these.

-Authors describe interindividual variation of GalNAz incorporation in terms of “relatively similar” or “larger variation” in each cell line. Statistical significance should be indicated in terms of an analysis of variance.

Response: We added statistics to the text: ‘There was no statistically significant difference in the level of GalNAz incorporation between the cell lines (p = 0.11). In the RTgill-W1 *in vitro* membranes, the level of incorporated GalNAz was relatively similar between membranes (Standard deviation, SD: 9.88, Figure 2C), while a tendency to larger variation between membranes was observed among the CHSE *in vitro* membranes (SD: 20.98, two sample F-test: p = 0.12, Figure 2F).’

Furthermore, we added a more detailed description in the figure legend, in which we also included the variance: ‘The quantified fluorescence intensity data sets for both cell lines passed the Kolmogorov-Smirnov test for normality. The sample variances (SD^2^) were 97.55 and 440.11 for RTgill-W1 and CHSE-214, respectively. The fluorescence intensity did not differ between RTgill-W1 and CHSE-214 (T-test, p = 0.11).’

-L430 and following: Authors could include a comment on the need of semi-wet interfaces for fish in vitro membranes, since in vivo fish membranes remain immersed in the natural environment.

Response: we have expanded the section so it now reads ‘Although fish are submerged in water most of the time we considered trying this method to ‘trick’ the cells to increase mucus production, however, the effects of a semi-wet interface with mechanical stimulation on mucin production could not be explored in the fish *in vitro* model here, as a rocking platform increased the incubator temperature by 12 °C, resulting in a suboptimal environment for the fish cells. Instead, the fish cells were cultured submerged in medium both on the apical and basolateral sides.’

-L464-477: Have authors some hypothesis on the differential sialylation and fucosylation of the analyzed mucins compared to fish gill mucins? Do they have any suggestion in order to modulate the glycosylation pattern so that their mucin model may better replicate the in vivo pattern?
In the same line, PAS+ goblet cells with neutral mucins are usually present in fish mucosal epithelia. Authors could also discuss the absence of those in their in vitro model.

Response: It is quite common that cell lines do not exactly mimic the glycosylation present in the epithelial site of origin, for example glycans can be truncated. We have added ‘If fucosylation is an important feature for planned experiments, it is possible to transfect cell lines with glycosyltransferases, including fucosyltransferases, to obtain a required glycoprofile, and included two references for this (<https://www.nature.com/articles/s41467-021-24366-4>

https://www.ncbi.nlm.nih.gov/pmc/articles/PMC1602278/) to the discussion section. In the part of the discussion pertaining to bacterial binding, we also added ‘That both cell lines carry epitopes *A. salmonicida* can bind to supports that they can be used to study host-pathogen interactions. For example, it could be interesting to investigate the role of specific glycans in host pathogen interaction by gene editing the cells for specific glycosyltransferases and compare the impact of presence/absence on pathogen binding and downstream host responses.’ We are not certain that PAS+ goblet cells are an abundant feature of gill epithelia in rainbow trout – In response to this comment we stained approximately 90 rainbow trout gills with PAS/AB, and only found rare PAS+ cells - we would say at least 95% of the cells were blue, very few were pink. We did not have any chinook salmon samples available, and did not find images of PAS+ by googling. Since we did not find clear evidence supporting that neutral goblet cells are a major feature of rainbow trout gills, we prefer to not write a discussion focusing on this. If the reviewer can point us to publications demonstrating that they are, we would be happy to incorporate them in the discussion, however, since we now have written about the possibility to gene edit the cell models to obtain different glycoprofiles and pointed to the lack of fucosylated glycans in the model, maybe this is sufficient?

-Authors do not consider the differential involvement of secreted and membrane-bound mucins in their study. Especially when mucins from cell lysates are analyzed, such different mucin types in addition to intracellular immature mucins associated to the Golgi apparatus, should be considered.

Response: We agree that what we analyzed was the glycan repertoire of the whole cell and not of individual mucins, or glycoforms of individual proteins. We do not deem it feasible to isolate different mucins separately for glycan analysis with the tools currently available for rainbow trout and chinook salmon.

Besides, some minor corrections should be implemented:
- L49: Please, change for “fish mucosal epithelia are”.

Response: we have re-written as requested.

- L108: Correct “RTgutGC”.

Response: the missing ‘t’ has been added, thanks for detecting this error.

- L166: Please, correct “The medium … was”.

Response: we have corrected to medium.

- L169: Correct “mucosal surfaces” to lower case.

Response: we have corrected to lower case.

- L204-234: Use µL for microliters.

Response: we have replaced the u with µ.

- L218: Describe what “conc HAc” is.

Response: we have written out ‘acetic acid’.

- L225: Shift to “control media”.

Response: we have shifted to ‘control media’.

- L251: Correct “MS/MS”.

Response: we have corrected to MS/MS.

- L310: Please, place “…, in appearance, …” between comas.

Response: we have added the requested commas.

- Table1: Empty cells should be filled with 0 or dash.

Response: er have added the requested dashes.

- L393: Please, rewrite. E.g. “…their relative abundance was estimated…”

Response: we have re-written as requested.

- L411: Please, rewrite. E.g. “… is incorporated similarly in both cell lines and Alcian blue staining was also similar in both”

Response: the suggested sentence does convey the meaning that we tried to convey. Therefore we did not rewrite as suggested.

- L412: Please, change “which were all”.

Response: we have re-written as requested.

- L419: Correct “models that mimic”.

Response: we have re-written as requested.

- L420: Correct “often result in”

Response: we have re-written as requested.

- L425: Please, delete “of mucus”.

Response: we deleted as requested.

- L500: Correct “models present”.

Response: we removed the s as requested.

Reviewer #2: The manuscript title “Mucin Producing In vitro Fish Mucosal Surfaces as a Model for Studying Host” by Quintana-Hayashi and colleagues is about the possible use of fish cell lines as in vitro mucosal surface models. These models pretend to be an alternative to in vivo experiments and be a model to investigate the effect of pathogens and modulatory components on mucin production in fish.
I found this article really interesting and a good alternative to reduce the number of fish in in vivo experiments. However, I found the manuscript difficult to read because the material and methods are not well explained and therefore the workflow is confusing. In addition, I missed a proper control in many experiments and statistical analysis. For all of these reasons, I consider that is needed deep changes in order to be published.
1-I do not know the protocol that authors used to differentiate fish cell lines into mucus producing cells. In material and methods it seems that only with culturing the cells in snapwell cell culture for 2 weeks and after that with the inhibitor DAPT and IWP-2 is enough but in the results is suggested that authors have used other methods to differentiate cell for example: line 278-282 “We started by investigating morphology, adherence, and mucin production in the salmonid cell lines RTgill-W1, CHSE-214 and SSE-5 under standard conditions as well as under conditions that we have used in the successful development of mammalian in vitro mucin producing mucosal surfaces from epithelial cells, including treatment of the cells with goblet cell differentiating media and prolonged culture ”. Please clarify these issue.

Response: We have re-written the methods section Chemical stimulation of the fish cells to make it clearer. We have also shortened the results section to make it clearer (line 147).

2-The workflow of the experiments is confusing, for example in the results of the sections: The metabolic label GalNAz is incorporated into both RTgill-W1 and CHSE-214 based in vitro membranes, O-glycan analyses from CHSE-214 and RTgill-W1 and A. salmonicida binds to RTgill-W1 and CHSE-214 cells. I do not know the protocol that authors used to differentiate fish cell lines if it is the same that in the section RTgill-W1 and CHSE-214 cell lines form an adherent layer and produce mucins. Please clarify this issue. I suggest to draw a schema in order to clarify how the experiments are performed.

Response: Please refer to the methods sections where all methods have been described, which we have now clarified. We have also added more information on the experiment to the figure legends to make this clearer.

3-Material and methods are incomplete; I do not know how the experiments are made and I missed commercial references of many reactives in many sections. In addition, I did not find the microscope use for the images taken in figure 1 and Figure2. In figure 2 C and F, I do not know how authors obtain these data and there are not properly explain in the legend. Moreover, in many sections of material and method, authors said for example aliquots were dot plot, cell lysate aliquots or paraffin embedded sections… but they do not indicate which samples were analyzed and therefore makes more difficult to understand the experiments that they perform.

Response: We have gone over the methods section to ensure that all specialty chemicals have a commercial reference. We have added the information that the images were taken with an Eclipse 90i microscope (Nikon) to the figure legends. We have added the information about the quantification briefly to the figure legend of Figure 2 and in the methods section in more detail. We clarified this where it was unclear. Since there is a section in the methods describing how the cell lysate was made, we think it is clear what the cell lysate is and have added cell lysate to ‘aliquots’ to clarify.

4- In addition, I missed a proper control of many experiments. For example, in figure 2 I do not understand why the not labeled membranes are stained. I must include a positive and negative control to compare the results obtained and include also in C and F graphs. In figure 3 andfFigure 4 I would include a control of CHSE and RTGill without differentiate as a proper control in addition to the media control.

Response: In figure 2, the non-labelled membranes are the negative control. To clarify this, we added the words ‘negative control’ to the figure legend. The labelled membranes are very bright in comparison, which is evident without quantifying the negative control (in which the red stain is virtually absent, the blue stain is DAPI-stain of nuclei). The TAMRA (tetramethylrhodamine) glycoprotein detection kit was used on both non-labelled and GalNAz labelled membranes. We have added this information to the methods section to clarify this issue further. The cell lysate used for figure 3 and 4 is without differentiation on membranes as we need much material for these assays – please see the description of cell lysate in the methods section. The media control is the important control as there are glycans also in the media, the purpose is thus to show that the cell lines produce molecules that the bacteria bind to, and those are not a contaminant from the media.

5- In figure 1 and Figure2 authors should include in both legends the magnification of these pictures and the number in the scale. In figure 2 please include statistical analysis of the graphs.

Response: In the figure legends of both figures it states: ‘Scale bar = 30 µm.’ We have added statistical analysis to Figure 2 ‘The quantified fluorescence intensity data sets for both cell lines passed the Kolmogorov-Smirnov test for normality. Results of a two sample F-test for equality of variances indicated that there is a non-significant (p = 0.12) large difference between RTgill-W1 (standard deviation, SD = 9.88) and CHSE-214 (SD = 20.98). The sample variances (SD^2^) were thus 97.55 and 440.11 for RTgill-W1 and CHSE-214, respectively.’ We have also added a statistics section at the end of the methods section and included the outcome of the statistical analyses in the results text.

6-The binding assay in my opinion is not enough to confirm this issue. authors should include more experiments to confirm the binding.

Response: We were first very surprised about this comment, but then realized that the figure in the review site is very dark and barely visible – this is in stark contrast to the very clear binding signal in the original image. We have contacted the journal to sort this out. The only point we make with the figure is that both cell lines produce molecules that the bacteria bind to. The binding signal is very clear to the cell lysate wells (green fluorescence) and there is no binding signal to the media or PBS wells from the slot blot. The presence of a strong green signal to the slot blot wells with cell lysate is very clearly stronger than the negative controls.

7-The results showed in table 1, in my opinion, the spectrum of the peaks of the chromatogram of O-glycans should be included in the manuscript. I do not understand why some samples are dot blot and others by gel band, please explain properly.

Response: the spectra are available, please see in the methods section ‘ Raw MS data on *O*-glycan analyses are available on glycopost (https://glycopost.glycosmos.org/) using the project ID: GPST000334 (CHSE-214) and GPST000335 (RTgillW1). MS/MS data with tentative structures are available <https://unicarb-dr.glycosmos.org/references/523>.’

We have clarified the information on this in the results section under ‘O-glycan analyses from CHSE-214 and RTgill-W1’, we fist analyzed O-glycans from cell lysates and then put the same material onto SDS-page to ensure that the glycans described were from the cells and not a contaminant from the media: ‘Although the cells were washed before lysis, the media the cells were grown in was also analyzed to ensure the glycans described were from the cells and not contaminants from the media. The media contained NeuAca2-3Galβ1-3GalNAcol, NeuAc-Gal-(NeuAc-)GalNAcol and Gal1HexNAc1GalNAcol, albeit at different proportions than the cell lysates (Table 1). To verify that the glycans originated from the cell lines (i.e. not a contaminant from the media), reduced and alkylated proteins from cell lysates were separated with SDS-PAGE for large proteins, and the gels developed with Alcian Blue, which stains for acidic glycans (Figure 3).’

d b8- I found some confusing sentences that I detaile elow:
-line 80-82” A decreased secretion could lead to an increased amount of mucus in the cells, conversely, an increased secretion could lead to a decreased amount of mucus in the cells”. please clarify.

Response: We have now clarified the section so it reads ‘In a situation where the mucin production rate is unchanged, a decreased secretion could lead to an increased amount of mucus in the cells, conversely, an increased secretion could lead to a decreased amount of mucus in the cells. The amount of mucus within the goblet cell is thus not strongly linked to mucus production rate.’

-line 461-463” the lower inter-individual variation and more distinct GalNAz staining of
the RTgill-W1 in vitro membranes most likely makes 462 this a better model for studies comparing effects of different conditions/treatment on mucin production”. I do not understand the sentence “more distinct GalNAz staining”, in the graph seems that there are not much variation in the fluorescence intensity. Please clarify this sentence.

Response: We have removed the comment on more distinct staining in the discussion and added standard deviation and variance analysis to the results section. We also re-wrote the section to ‘A lower number of replicates are thus likely to be needed when using the RTgill-W1 *in vitro* membranes compared to CHSE-214 for studies comparing effects of different conditions/treatment on mucin production.’

-line 474-476”. Although the majority of the rainbow trout mucins are sialylated in line with the results from the RTGill-W1 mucin glycans, approximately 30% of the glycans are sialylated (6), a feature we did not detect among the RTGill-W1 mucin glycans”. It is contradictory the information in the sentence, first authors observed sialylated mucins but at the end of the sentence said they did not detect. Please clarify. In addition, line 487-488 “The high abundance of sialylated glycans is likely the reason that Aeromonas salmonicida bound to both cell lines”. Please clarify.

Response: we apologize for the typo, we have corrected the sentence to ‘Although the majority of the rainbow trout mucins are sialylated in line with the results from the RTGill-W1 mucin glycans, approximately 30% of the rainbow trout gill glycans were fucosylated (6), a feature we did not detect among the RTGill-W1 mucin glycans. ‘

We assume that the reason that the reviewer thought the sentences *‘Aeromonas salmonicida* binds to NeuAc, especially when linked via a 2,6 linkage (7, 14), which was present in both cell lines. The high abundance of sialylated glycans is likely the reason that *Aeromonas salmonicida* bound to both cell lines.’ were confusing might be that the reviewer was not aware that NeuAc is a sialic acid. We therefore added sialic acid to the first sentence to make this clear.

Minnor things


Line 33, DAPT define acronym.

Response: We have added the requested information.

Line 111-112 cldn3 and il-1β, il-6, il-8 and tnfα define acronym

Response: we have now defined the acronyms.

Line 154 WNT acronym

Response: Wnt is created from the names Wingless and Int-1. It is usually written as Wnt, and I think it might be confusing for readers to expand this.

Line 218, conc HAc define acronym

Response: we have now written out acetic acid.

Line 298 figure 1, I will specify to Fig 1A, F in order to reference the information which is included in the sentence.

Response: we have now added A, F.

Line 307 AB define acronym

Response: We have now written out Alcian blue.

line 367 the bottom, I believe that it is on the top. Please change it.

Response: It is actually just below the bottom of the well as it states in the manuscript. Maybe the confusion stems from that the well is at the top of the gel? We added ‘loading’ as in loading well and hopes this makes it clearer.

-Indicate at least for the first time define h as hours

Response: we have now written out hours (h) the first time in the manuscript.

-Line 426 HT29-MTX-E12 define acronym and the specie of the cell line

Response: we have now added human to ‘colonic HT29-MTX-E12 cells’. The letter combination is the name of the cell line.

-Line 508 Neu Ac define acronym

Response: we have now written out N-acetylneuramic acid.

Reviewer #3: *General comments: The current manuscript proposes the use of fish cell lines as a potential model platform for evaluating host/pathogen interactions on mucosal surfaces by mucin production.

I think that the strengths of the study are the methods to differentiate cells and induce mucin production, which are quite clear and described in detail. Furthermore, considering the historical lack of tools and platforms at the phenotypic level in fish, this study also contributes to the in vitro characterization of the immune response in epithelial barriers. However, regarding its weaknesses, I think that a more quantitative or semi-quantitative evaluation of the different parameters evaluated was lacking, especially related to the production of mucins (Figure 1) and the capacity of the cell lysates to bind A. salmonicida (Figure 4). Finally, before proposing final acceptance of the manuscript there are aspects to improve, which I detail in the specific comments:

*Specific comments:

Title: The first part of the title could change from "Mucin Producing In vitro Fish Mucosal Surfaces" to "In vitro Fish Mucosal Surfaces Producing Mucins". This would improve the understanding of the title.

Response: We have changed the title as requested.

Keywords: "cell line" should be added

Response: we have added cell line as a key word

Abstract:
-Line 23: After "prevent disease and reduce antibiotic use." There is a sentence missing that connects this idea with the "Fish epithelia secrete mucus". For instance "To deal with this problem, fish epithelia that secrete mucus, which is mainly composed of highly glycosylated mucins, are a potential target for evaluation."

Response: we have now rewritten this section into ‘Mucus covered mucosal surfaces are the first barriers pathogens encounter. Mucus, which is mainly composed of highly glycosylated mucins, has the potential to contribute to disease prevention if we can strengthen this barrier.’

-Line 25: After "pathogens encounter." add "Therefore," the aim.. .

Response: we added therefore as requested.

-Line 41: Change "These models thus" to "Thus, these models"

Response: we made the requested change.

Materials and Methods:
-Line 138: Add "," after "flask"

Response: we added the requested ,.

-Line 175: In "H2O", the 2 must be in subscript.

Response: we corrected to H_2_O.

-SSE-5 should be eliminated from the manuscript since due to their problems in adhering, the authors did not carry out the tests with these cells nor did they obtain data.

Response: We deleted SSE-5.

-Line 259: Performing blotting to determine the binding capacity of cell lysates to A. salmonicida does not seem to be the most sensitive test or the one that allows the best analysis. Considering the capacities that the authors have described, the test should have been carried out in 96-well plates with adherent capacity to determine OD, this would have allowed a quantitative experiment with better comparisons between the samples.

Response: We were first very surprised about this comment, but then realized that the figure in the review site is very dark and barely visible – this is in stark contrast to the very clear binding signal in the original image. We have contacted the journal to sort this out. To clarify the purpose of the A. salmonicida binding assay, we added the sentences ‘That both cell lines carry epitopes *A. salmonicida* can bind to supports that they can be used to study host-pathogen interactions. For example, it could be interesting to investigate the role of specific glycans in host pathogen interaction by gene editing the cells for specific glycosyltransferases and compare the impact of presence/absence of specific glycans on pathogen binding and downstream host responses.’ to the discussion. We thus do not make a comparison between the cell lines, we conclude that the bacteria investigated bind well to both cell lines. Our opinion is that the slot blot based method is the best for determining binding to a whole cell lysate. In microtiter plates, small non-glycosylated proteins coat the plastic surface faster than the large mucins. Mucins are thus quite under-represented in microtiter based assays if not using purified mucins. To Isolate and purify mucins is a very time consuming process and requires much material, in the order of 1000-fold more material than normally used when using cell lines to obtain pure mucins. The method chosen here, attaching the contents of the cell lysates to a membrane allows mucins to attach to the membrane. This method can actually be used for quantification of the infra-red signal, which we have done in other publications. However, there is no scientific need to measure if the bacteria might adhere slightly better to one cell line or the other– indeed the signal suggests that the level of binding is relatively similar between the cell lines. However, quantitative experiments could be interesting to investigate the role of specific glycans in host pathogen interaction by gene editing the cells for specific glycosyltransferases and compare the impact of presence/absence on pathogen binding and downstream host responses, as now included in the discussion.

Results
-Line 277: RTgill-W1 and CHSE-214 cell lines form an adherent layer and produce mucins. The authors, in addition to showing the images (and describing them), should perform a semi-quantitative analysis (in software such as ImageJ) of the intensity and coverage of the labeling between the different samples. This will allow comparisons between cell lines, inducers and mucin production to be better established.

Response: Fluorescence intensities were measured using NIS-Elements D v. 4.0 software and comparisons were made between cell lines as shown in Figure 2. For figure 1, it is our opinion that the photographs give readers a better understanding of the model and effects of treatments than what a graph could do. For example, for certain experiments a thin cell layer such as that present after 2 weeks of culture might be better than the multilayer present after 4 weeks.

-From 278 to 286: This paragraph is just methodology again, it should be moved to that section (modifying it).

Response: we have moved most of the section to methods and shortened it.

-From 327 to 331: Just methodology again, it should be moved to that section (modifying it)

Response: we have removed the section as requested.

-Seccion "The metabolic label GalNAz is incorporated into both RTgill-W1 and CHSE-214 based in vitro membranes". In figure 2, C and F should be merged and thus statistically evaluate the fluorescence intensity between RT-gill and CHSE214.

Response: There is no statistically significant difference in fluorescence intensity between the two cell lines (p = 0.11). We have added this information to the results section and the figure legend for Figure 2. We have moved the graphs so they are side by side as requested.

-From 348 to 350: Methodology again, it should be moved to that section (modifying it).

Response: We removed the information as requested.

-Line 396: "A. salmonicida binds to RTgill-W1 and CHSE-214 cells" should be modified to the ability of the cell lysates to bind to the bacteria. For example: RTgill-W1 and CHSE-214 cell lysates binds A. salmonicida".

Response: we changed the sentence as requested.

Discusssion:
Lines 485 and 487: Change "Aeromonas salmonicida" to "A. salmonicida"

Response: we changed as requested.

Figures
In general, the resolution of the manuscript images that contain cells is low (even in the downloaded versions). This must be improved to achieve publication of the study.

Response: This must have to do with the review site, as the images uploaded are high resolution. We have had this experience with PLoS ONE before, and found that this is something that can be corrected at the proof stage. We have contacted the journal to sort this out.

References: Authors should consider reducing the percentage of self-citations (currently 60%). Of the 45 citations in the text, 27 are from one of the authors involved in this study. Multiple references in the same sentence should be eliminated and others from different groups incorporated, for example in the introduction.

Response: we have incorporated six additional references as requested and removed multiple references in three places. The reason for the self-citations is that we are the only group who have measured mucin production and glycomics in salmonids which are the major themes to the manuscript and the in vivo reference material we compare the data too. Furthermore, we have also tested methods for cell differentiation that we developed previously. Thus, we consider these the most relevant papers on the topic. We have removed several of the multiple references now. However, multiple references to a sentence are relevant when there are several studies that contributes to the statement, and multiple references for single sentences are common in PLoS journals. If the editor thinks we should not have multiple references after statements we are willing to make this change, but as this is common practice we think this might be an individual preference.

**reviewer 4** (the additional reviewer that arrived as an attachment)

1. Use full form of RT and mention room temperature for country where the experiment was performed. (Line no – 194)

Response: we have written out room temperature (approximately 20 °C) as requested

2. Typographical error in writing 14000 kD. (Line no – 224)

Response: we have corrected this now.

3. Typographical error in writing 4 °C. (Line no – 228)

Response: we have added a space.

4. Ultimate3000, is this correct way to write or typographical error? (Line no – 236)

Response: we have corrected to UltiMate 3000

5. autosampler, is this correct way to write or typographical error? (Line no – 236)

Response: this is correct.

6. Typographical error in writing 35 %. (Line no – 244)

Response: we have added the space.

7. Typographical error in writing 20 °C. (Line no – 257)

Response: we have added the space.

8. OD600nm, is this correct way to write or typographical error? (Line no – 268)
9. Response: have now written this out in full: optical density (600 nm)
10. Use standard format while writing Alcian blue in the entire manuscript.

Response: we have changed to Alcian blue in the entire manuscript.

11. interindividual, is this correct way to write or typographical error? (Line no – 337)

Response: to avoid confusion, we have changed to ‘larger variation between membranes.

12. nonderivatized, is this correct way to write or typographical error? (Line no – 349)

Response: we have removed this sentence.
